# Caregiver Perceptions of the Potential Utility of a Specialized Family Peer Program for Anxious Youth

**DOI:** 10.1007/s10826-025-03173-1

**Published:** 2025-10-18

**Authors:** Jesslyn M. Jamison, Michal Weiss, Danielle R. Adams, Sophia Young, Megan Brady, Wanda Cummings, Crystal Karenchak, Emily M. Becker-Haimes

**Affiliations:** 1 Department of Psychiatry, University of Pennsylvania Perelman School of Medicine, Philadelphia, PA, USA; 2 The Leonard David Institute of Health Economics, Colonial Penn Center, Philadelphia, PA, USA; 3 Hall Mercer Community Mental Health, University of Pennsylvania Health System, Philadelphia, PA, USA; 4 Center for Mental Health Services Research, Brown School of Social Work and Public Health, Washington University in St. Louis, St. Louis, MO, USA; 5 UPMC Western Psychiatric Hospital, Youth and Family Training Institute, Pittsburgh, PA, USA; 6 Family Policy and Engagement Consultant for PA Care Partnership, Commonwealth Towers, Harrisburg, PA, USA

**Keywords:** Childhood anxiety, Caregiver, Family peer, Access, Treatment

## Abstract

Caregivers of anxious youth face significant and unique barriers to accessing evidence-based care for their children. Family Peer (FP) support is a promising strategy used within children’s mental health services to address barriers to accessing and engaging in mental health treatment. However, it has not yet been developed or tested specifically for caregivers of anxious youth. This study gathered perspectives from caregivers who scheduled but did not attend their initial appointment at a specialized pediatric anxiety clinic in a Northeastern city in the United States. We purposely recruited caregivers with either public (Medicaid) or private commercial insurance (provided through an employer). Twelve recruited caregivers completed qualitative interviews to report on barriers and facilitators to accessing mental health services and their perceptions of the potential utility of FP support. They also provided brief quantitative ratings of the utility of different types of support that FPs can provide. Caregivers described significant barriers to accessing care. They shared positive reactions and interest in engaging with FP support. They expressed importance of the FP having a child of similar age and sharing diagnostic features. Themes differed between Medicaid-insured and commercially-insured families. Overall, Medicaid-insured families expressed more interest in engaging with FP support in interviews and provided significantly higher quantitative ratings of the potential utility of FP support for four of six types of support queried. We also observed differences by insurance status for other key themes, including preferences for how they would want the FP to support families and descriptions of how FP support might be most helpful. Results of this study will be used to guide the development of an FP program specifically tailored for caregivers of youth with pediatric anxiety disorders. We also discuss the broader implications of these findings related to the potential of FP support for pediatric anxiety.

## Background

Anxiety disorders often emerge in childhood and are associated with significant impairment in daily functioning across domains (Muroff & Ross, 2011). Though anxiety disorders are among the most common mental health disorders in the U.S. (Bitsko, 2022), only one in five adolescents are able to access anxiety treatment (Merikangas et al., 2011). Even fewer will access the gold-standard, exposure-based treatments thought to be critical to treatment success (Higa-McMillan et al., 2017). Treatment access for internalizing disorders is notoriously challenging compared to other mental health concerns; this is due in part to underutilization and low availability of exposure-based treatment (Higa-McMillan et al., 2017; Hofmann et al., 2012; Wolitzky-Taylor et al., 2019). Connecting youth with anxiety disorders to evidence-based treatment and timely access to diagnosis is critical for preventing additional psychopathology and impairment in adulthood (Saavedra et al., 2010).

Families seeking treatment for their child’s anxiety encounter significant barriers to accessing treatment (Crouch et al., 2019; Frank et al., 2020; Reardon et al., 2017, 2018; Salloum et al., 2016). These include pervasive structural barriers, such as long waitlists, costs, and lack of transportation as well as attitudinal barriers, such as stigma and negative perceptions of mental health treatment (Hansen et al., 2021; Owens et al., 2002; Reardon et al., 2017; Zifkin et al., 2021). There also are unique barriers specific to a child’s ability to receive anxiety specific services. For example, caregivers of anxious youth endorse difficulty with knowing when anxiety exceeds what is developmentally typical (Reardon et al., 2017; Salloum et al., 2016) and how to differentiate anxiety symptoms from other difficulties (Crouch et al., 2019; Frank et al., 2020), which can delay the time it takes for caregivers to begin the process of seeking support. Families of anxious youth also endorse gaps in knowledge with respect to the importance of exposure therapy for treatment success and where to receive such services, often resulting in their children receiving ineffective therapies before they finally connect with a provider trained in exposure (Frank et al., 2020).

Finally, given the known genetic loading of anxiety within families (Meier & Deckert, 2019), it is perhaps unsurprising that caregivers also report a high degree of their own anxiety about the treatment seeking process and describe a high emotional toll associated with being uncertain about how to best help their child (Chavira et al., 2017; Frank et al., 2020). Barriers to accessing anxiety treatment may be amplified by factors that limit the availability of exposure-based cognitive behavioral therapy, or “exposure therapy” including underutilization of exposure therapy by providers working in routine clinical settings (Deacon et al., 2013; Whiteside et al., 2020). Consistent with these findings, qualitative studies with caregivers of anxious youth have highlighted the extreme perseverance required of families to access anxiety treatment for their child and the need to reach out repeatedly to providers to successfully access treatment (Crouch et al., 2019; Frank et al., 2020).

Taken together, the need for effective care to prevent negative sequelae of untreated anxiety disorders, the importance of connecting families to timely exposure therapy, and the unique barriers encountered by families seeking pediatric anxiety treatment call for tailored and creative solutions to address this population’s unique needs. In the broader literature, one proposed way to increase access to and utilization of mental health treatment is utilizing Family Peers (FPs) to help support caregivers seeking mental health treatment. FPs are caregivers with lived experience of having a child with mental health needs who are specially trained to support other caregivers. FPs have been used within child mental health services broadly and have shown promising results in randomized controlled trials for increased caregiver engagement with mental health services (Bearman et al., 2022), increased caregiver feelings of efficacy and empowerment (Kutash et al., 2011), and increased caregiver knowledge about their child’s disorder (Jamison et al., 2017). Studies also demonstrate that for caregivers of children with Autism Spectrum Disorder, connection with an FP significantly increased the probability of completing a diagnostic evaluation (Feinberg et al., 2016, 2021). FP support has also been found to decrease parental stress (Ireys & Sakwa, 2006; Jamison et al., 2017), improve coping with and acceptance of their child’s mental health needs (S. Singer et al., 1999), and increase parenting skills (Conn et al., 2018; Day et al., 2012). However, no program to date has developed or tested an FP model specific to families of children with anxiety disorders.

FP support is broadly used across the United States, and is reimbursable through publicly-funded insurance, in a majority of states (Schober & Baxter, 2020). Within the broader mental health literature, there is not a consensus in how FP support is delivered. This includes diversity of format (e.g. group, individual), frequency of meeting, and mode of meeting (e.g. phone, in person). However, some consensus exists in the content of FP support across programs and models. In a 2005 review of broad Family Peer programs, Hoagwood and colleagues found that across models, Family Peers focused on the provision of informational support, instructional support, emotional support, instrumental support, and advocacy support (Hoagwood, 2005). To date, the potential utility of these types of FP support specifically for pediatric anxiety have not been explored.

Tailored FP support may be well-suited to increase access to mental health treatment for caregivers of youth with anxiety. Several qualitative studies examining the experiences of caregivers seeking care for their child with anxiety indicate caregiver interest in engaging with peer support (Crouch et al., 2019; Frank et al., 2020), especially when they are on waitlists prior to beginning treatment (Crouch et al., 2019). However, no study to date has gathered feedback from families on their interest in and their perceptions of the utility of FP support tailored for caregivers of youth with anxiety, nor how FP support maps onto identified barriers and facilitators to care specific to accessing anxiety treatment. To address this gap, we conducted qualitative interviews with caregivers of anxious youth who attempted to seek specialized anxiety treatment to learn their perceptions of the barriers, facilitators, and potential utility of FP support. We were particularly interested in learning from caregivers who struggled to engage with anxiety services for their child, as these are families who have been historically the most difficult to engage in services and are the most likely to benefit from novel strategies such as FP support. In addition to determining acceptability, a secondary goal of this data collection effort was to inform the development and tailoring of an anxiety-specific FP program. Preliminary work to learn from caregivers most likely to benefit from such a programs (i.e., at high risk for not engaging successfully with evidence-based treatment) can guide program development to optimize its eventual effectiveness.

At the time of data collection, we had not yet developed an FP program specifically dedicated to specialty anxiety treatment for youth. We report here on initial work conducted to determine whether specialized Family Peer for anxiety would represent an acceptable option to families who had not engaged in case at the clinic and whether it had the potential to map onto identified barriers to care for these families to guide program development.

## Method

### Setting

The study took place at a specialized pediatric anxiety mental health clinic located in a Northeastern United States city. It is the only pediatric specialty clinic in the city dedicated to serving low-income youth with publicly funded (i.e., Medicaid) insurance plans. Medicaid insurance, or medical assistance, refers to public insurance in the United States, which is provided to families either based on family-income level or child level of mental health need. Commercial insurance is typically provided through a caregiver’s employer. About 60% of families who come to the clinic are covered by Medicaid. The clinic also serves families with the hospital employee commercial insurance plan. At this clinic, families with other commercial insurance plans pay for services out-of-pocket because their insurance is not accepted.

### Participants

Participants were twelve caregivers who sought treatment at the clinic between March 2021 and July 2023. To recruit, we reached out to families who had sought care at the clinic during this time frame but had not attended their initial appointment. Additional details on participants who received outreach are provided below. Of note, participants largely sought services during a time of markedly increased demand for youth mental health services (Benton et al., 2022) in the wake of the COVID-19 pandemic (3 sought treatment in 2021, 7 in 2022, and 2 in 2023). On average, participants were on the waitlist to receive care for 103 days (approximately three and half months) before their intended intake appointment, with a range from 56 days to 162 days. Caregivers were eligible to participate in the study if they completed an initial screen and scheduled an intake appointment but ultimately did not up attend that appointment. Due to study team constraints, eligible participants were also required to be English-speaking. Given that our clinic serves a primarily Medicaid-insured population, and historically these families have faced additional barriers to accessing care (Koob et al., 2023), we purposefully sampled a mix of both Medicaid-insured families (*n* = 5) and commercially-insured/self-pay families (*n* = 7). Participant demographics are shown in Table 1.

### Procedures

All study procedures were reviewed and approved by the University of Pennsylvania Institutional Review Board. Individuals who met inclusion criteria were invited via email and phone to participate in a 30-minute semi-structured qualitative interview to share their perspective on accessing services for their child. Fifty-four potentially eligible caregivers who did not attend their scheduled intake appointment for their child were contacted about the study using both email and phone contact, when available. Of those, three did not have a correct contact number, one was Spanish-speaking only, 14 did not respond, and 22 declined because they were either too busy or not interested. Two participants expressed initial interest but did not attend their scheduled consent call and interview. Interested participants provided verbal consent to participate in the study and were compensated $25 for participating. Participants were informed that their participation in the interview would not affect their ability to receive services at the clinic in the future. Interviews were conducted by research staff uninvolved with clinic scheduling and intake assessments (authors JJ and MW), and interviewers and participants were not known to each other prior to the interview. The study team had ongoing conversations to determine whether code saturation and meaning saturation (Hennink et al., 2017) was reached both across the full sample and by insurance status. Study team members met after every few transcripts to discuss whether new themes were emerging. While we did not set out initially to specifically recruit commercially insured family, lack of code saturation required us to surpass an initial recruitment target of 10 to include 12 individuals representing a mix of Medicaid and commercially-insured families in order to gain saturation within each group.

During the interview, participants were asked about barriers and facilitators to accessing anxiety-focused mental health services for their child, past experiences navigating mental health services, and their perceived utility of an FP program. To further contextualize qualitative results, caregivers also verbally answered a series of brief quantitative questions asking them to rate the potential utility of six different types of FP support that are commonly delivered (Hoagwood, 2005) on a Likert-scale from 1 (*not helpful*) to 10 (*helpful*) (See Appendix 1 for full interview guide). Additional contextualization or spontaneous elaborations on Likert scale ratings were included in qualitative analysis and examined for common themes. All qualitative interviews were audio-recorded, transcribed and deidentified by members of the study team for analysis.

### Data Analysis

Using a thematic analysis approach (Clarke and Braun, 2017), the research team followed six recursive phases: (1) data familiarization; (2) code generation; (3) theme construction; (4) theme review; (5) theme definition and naming; and (6) final conceptualization and writing. Author JJ drafted the initial codebook using deductive coding based on a priori areas of interest and included codes about initial reactions to FP support, helpful aspects of FP support, and personal characteristics of an FP that caregivers would want to know about. Codes addressed barriers and facilitators to accessing mental health services, perceptions of telehealth, past experiences working with mental health navigators, perceptions of utilizing a family peer support service model, and suggestions for clinic improvements. The codebook was iteratively revised based on transcripts to include more detailed descriptions of each category as well as allowing for inductive coding where new codes emerged directly from the data, allowing for emergent themes and patterns. For example, the decision to analyze by insurance status or divide barriers into subtypes arose from inductive methods.

We used the Health Care Access Barriers Model (Carrillo et al., 2011) to categorize and conceptualize modifiable barriers to accessing mental health services identified by participants. Specifically, we used three categories for barriers: (1) *Structural* - barriers related to institutional and organizational factors, such as a lack of availability of providers, lack of transportation or childcare, long wait times, or difficulty navigating the mental health system, (2) *Cognitive* - knowledge and communication barriers such as stigma, health literacy, or availability of providers who are culturally competent, and (3) *Financial* – barriers related to the cost of care and health insurance, whether it be a lack of comprehensive coverage or administrative burdens implemented by insurance (e.g., preauthorization). These barriers have been associated with decreased screening and reduced engagement with treatment, ultimately leading to poorer mental health outcomes and health disparities (Carrillo et al., 2011).

The codebook was reviewed and refined by all study team members and then applied to the first four transcripts by authors JJ, MW, SY, and MB. To establish reliability, the first transcript was coded by four coders (JJ, MW, SY, MB) to establish consensus codes. The second transcript was coded by the first author and two primary coders (MW and SY) . Any sources of disagreement were discussed. Once the codebook was finalized and consensus established for the first two transcripts, two primary coders (MW and SY) each independently coded all 12 interviews and then met together to discuss and resolve any discrepancies. Kappas for primary coders ranged from 0.87 - 0.93 prior to discrepancy resolution. Author JJ weighed in on final decisions when coders disagreed. All transcripts were coded by two members of the team, and consensus was reached on 100% of codes. The final codebook is included in Appendix 2.

The full study team reviewed coded qualitative interview content to allow for synthesis of main themes. Author JJ conducted a primary thematic analysis of responses within each code. Responses were examined in two ways: 1) all together to examine overall codes and 2) by insurance status (Medicaid versus commercial insurance). For each code, a second coder independently provided a thematic summary of codes common to both groups and unique themes by insurance status. The two coders then discussed their thematic summaries and any discrepancies in summaries or differences in themes noted were discussed and resolved. Study participants were not given transcripts to review nor the opportunity to provide feedback on the findings. However, they could indicate whether they wanted to learn about study findings once they were published. Participant responses included in our results are denoted by their participant ID number (e.g. P3).

Prior to presenting findings, we want to acknowledge our positionality as a largely female and White identifying team. We have a vested interest in furthering FP programs and increasing the quality of care and the reach of evidence-based practices. We bring multiple lenses as a team including non-clinicians, clinicians, and FPs. The first and last author also bring the perspective of clinicians who work within the specialty mental health clinic from where participants were sampled. Therefore, an understanding of the clinic and FP support were represented in our analysis of results. To minimize bias, the two primary coders for this study who double coded all transcripts were individuals without prior knowledge of FP support nor direct relation to the clinic. However, group discussions about data reflected the broader range of perspectives.

Finally, quantitative data analysis consisted of independent samples t-tests to look at differences in perceived FP support ratings between Medicaid insured and commercially insured families. Quantitative results were used to further explore and validate themes identified in qualitative responses. We report below first qualitative then quantitative results. Key qualitative themes are reported below; codes from the codebook that did not generate substantive responses or can be subsumed under another code are reported as such.

## Results

### Barriers to Attending Initial Appointment

#### Structural barriers

Across insurance types, caregivers endorsed structural barriers as the main barrier to attending their initial appointment. Specifically, they identified length of time on the waitlist as a primary barrier. Additionally, caregivers shared that they had to call many different clinics and organizations and be added to multiple waitlists to access care. As one family explained, “I was trying to get help all the way around, whoever was able to get me an appointment I was willing to go” (P2). Though the waitlist was a common primary barrier to attending an initial appointment, the impact of this barrier differed by insurance status. Families with commercial insurance generally indicated that they did not attend an initial appointment because they were able to schedule with another provider who could see them sooner and took their insurance. Medicaid-insured families indicated that length of time elapsed on the waitlist (upwards of three months), being on various waitlists at multiple clinics, and the presence of multiple external stressors interfered with their ability to keep track of their scheduled initial appointment. One shared that even though they received a reminder for the appointment, the reminder appeared generic from the hospital system, and they were not certain what the appointment was for. As one caregiver whose child was covered by Medicaid described:

I know a lot of places I called- it was- the waitlist was like six months out and it was long, and I know at first it was long, when I called, but then I got a call-that’s why I didn’t think that I missed it, I didn’t know what it was for, I didn’t think- make my own reminder or whatever, because I would- he’s on a waitlist for a lot of stuff, so just like, I would’ve been able to bring him in and I could’ve gotten the ball rolling faster, if I would’ve, just knew what it was- I don’t know. (P2)

Another shared the impact of calling so many places and losing track of where they were on waitlists “because I was calling up places and trying to set things up” (P2). Other Medicaid-insured families alluded to overall stress and the difficulty of “juggling a lot” (P12). As they described:

[…] I think that most parents if they miss the appointment 9 out of 10 times it’s because life is crazy you know and I don’t think it’s like hey cause if they took the time to make the appointment they wanted to go, but then life gets kind of crazy not crazy in a bad way, but you know what I mean. (P12)

While caregivers across the sample endorsed structural barriers related to transport and making appointments fit within their life and schedule, Medicaid-insured families also cited additional structural barriers as primary barriers to care. This included housing instability and difficulty fitting in appointments during their work hours. One caregiver shared about the challenge they faced making the appointment fit within their and their family’s work schedules, as well as the difficulty they experienced logging onto the clinic’s online system from work:

I had no ways. I work all five days, so when I initially set the appointment, I was initially just gonna take an early break. And it was on [telehealth]- I was gonna go on [the telehealth platform] with me and my son. But when I realized I wasn’t able to do that. At the last minute, I tried to get my mom to do it while she was at home. But she had training. (P5)

### Cognitive barriers

Across insurance types, caregivers shared that their perceptions of their child’s symptoms influenced the way that they sought care and their decision or ability to ultimately not attend their scheduled intake appointment. Here, we also observed differences by insurance type. For commercially-insured families, these barriers largely presented as caregiver perception of acuity of child symptoms that required immediate support. This was an impetus to seek the first available specialized care for their child. These caregivers used terms such as “crisis,” “acute issue,” “desperate,” or “real urgency,” to describe their child’s need for care. As one caregiver explained it:

We were really trying to find immediate help and I would assume that most people and patients and family members of patients suffering from what seemed to be like pretty severe OCD being experienced for my daughter. Like we felt like we didn’t have a lot of time to wait for services. (P6)

While several commercially-insured families reported concerns about their child regularly missing school as a barrier to attending an initial appointment, one commercially-insured family tied this to concerns about child mental health symptoms. They described:

A lot of places, as I understood, are available at the same time my kids are in school. Well, the issue is you have school-related anxiety, is that you’ll have even more anxiety when you miss the class and you now have to make up work on top of it. (P10)

Alternatively, Medicaid-insured families often expressed more general uncertainty about whether to seek care for their child at all, concern about additional stress to their child with accessing a new service, or being unsure about whether a specialized anxiety clinic was the right fit. Caregivers shared diagnostic uncertainty about their child’s difficulties leading to ambivalence about pursuing specialty anxiety treatment. As one caregiver shared: “I guess because sometimes [the symptoms] are real mild and sometimes they’re real high so it’s confusing to me” (P12). Several Medicaidinsured families also shared how this influenced their own internal emotional processes. As one caregiver explained: “What with this being new to me- I was a little- I can say I was overwhelmed too, with you know, everything that was going on. [I was] Distraught- not distraught- but not coming forth, or not putting that much effort at first.” (P1)

### Financial barriers

We examined themes related to financial barriers while considering the unique nature of insurance coverage at the clinic. While across groups caregivers endorsed financial barriers to care, the types of financial barriers differed by insurance status. Commercially-insured families (who would have had to pay for the full cost of treatment out of pocket for services in this setting) reported the cost of the appointment as a barrier to attending an initial appointment and a desire to seek care at an alternative location that accepted their insurance. Medicaid-insured families mentioned cost associated with transportation as a barrier to attending their initial appointment.

### Facilitators to Attending Initial Appointment and Perceptions of Telehealth

We observed no differences in themes by insurance status for facilitators. Overall, caregivers reported positive attitudes towards addressing mental health needs and seeking mental health services. They expressed that family members and others in their community had similar positive attitudes. They often reported that they had shared that they were seeking mental health services with family members or that family members had encouraged or supported themin seeking care. As one caregiver shared about perceptions of mental health:

I raised [my children] to let them know this is who you are, and you’re actually extra special, because you’re always special, but with having mental health issues it makes you extra special, because I tell them that we think outside the box, or we think a certain way, and there’s nothing wrong with that. Because everyone has their own little special abilities, and this is ours. (P1)

Caregivers also identified the clinic location within the city as a facilitator to accessing treatment. Additionally, though most caregivers noted that telehealth was not a preferred mode for delivering therapy to their child, they characterized the availability of telehealth as a facilitator to accessing treatment for their family. As one caregiver explained:

I’m not saying there’s anything wrong with telehealth because telehealth actually has helped me, you know I said- because I physically, you know, cannot make it to every appointment, and telehealth has actually helped me in those times. So I think it was great to-for people to come up with the telehealth thing. I think that was a great idea. (P1)

Positive experiences with the scheduler were also noted as a facilitator to accessing treatment. For example, as one caregiver shared: “No, I mean they were very kind, and if I’m remembering, they said they would call me if there’s a cancellation. You know like there like we’re on it as soon as we can, we’ll call you and I believed them” (P7). One family identified their family’s lived experience with anxiety and OCD as a facilitator to seeking care because they were able to identify their child’s symptoms and need for support:

So my wife also has some OCD. So because of that we kind of understood some of the symptoms that my daughter was having and some of the struggles. Had it not been for my wife, I don’t think that I would’ve understood it. So that was- so I think that maybe helped us a little bit. (P6)

### Past Experiences with FP Support or Case Workers

No caregiver in our sample reported any previous experience with FP support. Some did report previous experiences with case worker support in navigating child mental health services; past experience did not vary by insurance status. Some caregivers described these interactions as helpful, while others reported they had difficulty getting in touch with their case workers or had only brief interactions. Many caregivers described having to navigate the system on their own, or “do it myself.” As one caregiver described, “I had to go on my own and do research on my own and try to you know, do things on my own. I wish I did have that support […]” (P1). Another described the process of trying to apply the knowledge they gained through helping other family members but still feeling like they needed additional support: “I’ve dealt with mental health things like throughout family, but I think that from what I’ve gathered I don’t know if I picked up key points” (P 12).

Unique to responses from commercially-insured families, caregivers shared connections they successfully made to other supports, whether that be to other caregivers who had navigated similar experiences with children of their own or from professional organizations:

I just am lucky to have found some friends who’ve had kids with similar needs and so, they have helped me just personally and I think that’s been a really huge help. I think having someone in the system that you’re specifically looking for- they were able to help you with that specific program. I think that would do wonders for sure. (P11)

Another caregiver shared about connections to additional supports:

My wife, I know, was in contact with a lot of different people involved with mental health and anxiety and OCD disorders. I don’t know the extent of exactly who she was communicating with, but I mean basically she was looking for somebody that could help her find appropriate services. So I know like she talked to a few different organizations in [location] and stuff, but I don’t think she really ended up getting what she wanted out of any of them. They tried to help, but like there just wasn’t a lot of services that we thought were appropriate that were available to us locally. (P6)

### Initial Reactions to FP Support

Across the full sample, caregivers shared positive initial reactions about the possibility of receiving FP support. Overall, caregivers reported high interest in receiving FP support, with most caregivers indicating it would be a useful service for them personally. Medicaid-insured families unanimously reported interest in receiving FP support for themselves. Interest in FP support was more mixed for commercially-insured families. Commercially-insured families who were not interested in FP support indicated that while they thought it might be helpful for others, they were only interested in support from a mental health professional at this time. As one caregiver shared:

I don’t think that is a service I would avail myself of. Sounds like a great service for some folks, it’s not something that I think I would particularly [want]. I think that what we are hoping for is a mental health professional to work with us as parents, and our daughter and give us like practical tips for helping her deal with her anxiety, and less looking for like the support that a peer-parent would offer. (P4)

Caregivers across groups highlighted the importance of the FP’s lived experience and interest in connecting with someone “who’s walking a similar road,” “who’s kind of traveled the same path,” or “has a similar parenting struggle to mine and someone who’s also trying to navigate the system.” They frequently expressed that this shared lived experience would help reduce isolation. As one parent expressed:

Yeah, I think because- like one knowing that you’re not alone- you’re not the only one whose kid is struggling, having the freedom to voice your own thoughts and maybe feeling the overwhelmingness you’re feeling […], and the exhaustion of dealing- you know with the anxiety all the time. I think it could be a normalizing piece of it, but also just to have a potential friend, maybe, who’s walking a similar road. I think could go a long way. (P7)

Caregivers also expressed the desire to learn from the FP’s lived experience and decisions that they made for their child. They highlighted the importance of gaining specific recommendations from the FP on where to turn for care whether learned from lived experience or from the FP’s training. As one caregiver shared about the idea of FP support:

I think that’s interesting. I think it depends on the peer parent, I think that if they’re educated and they’re like hey you can go here this is the best place, hey this place is super supportive, this place is crappy, you know over here is you know blah blah blah, something I didn’t know. I think that’s interesting. (P12)

Interestingly, among Medicaid-insured families, several families specifically expressed interest in the FP building a relationship with their child. The following excerpt exemplifies both the potential power of the FP’s lived experience and interest in the FP connecting with their child directly:

Listen, it relieves like a burden I have to thinking that-you know you’re not all alone, but you actually have this help with someone who knows what you’re going through and who can better assist you, and with a peer parent, to me, when it comes to my son, it’s- if it’s someone that- if he feels comfortable with this person, that’s just another support person that he has, which makes it even better for him because it’s someone else he can talk to, he can confide in, and he can get insight from. So I think that’s actually a great, great idea. Put me in the first one- if you’re doing it- make me the first one. (P1)

### Helpful Aspects of FP Support

Across insurance groups, caregivers indicated that they would be interested in learning both from the FP’s lived experience as well as the knowledge and training the FP had gained through other formats. In addition to the above noted impact of the FP on reducing feelings of isolation, caregivers expressed a desire to hear how FPs navigated their own experiences with their child, both in terms of services accessed as well as their own coping with their child’s needs. As one caregiver shared:

When I first found out what was going on- I was all over the place- my emotions and everything and I know it’s something new, so I would like to know like how they dealt with it. I just want him to be okay, I just want to [give him] whatever he needs, and this is new, so it’s just like I don’t even know the steps. So to hear somebody that went through it, and how they got through it, that would be a good thing. (P2)

Caregivers expressed that they would want to learn specific suggestions that had worked for the FP and their family. Caregivers wanted FPs to “give their insight, and what they did, and what worked for them,” “give me some direction on where to go and how to, you know, get him the help [my child] needs,” and learn from FPs to “avoid some pitfalls.”

We observed some differences here by insurance status. In discussing what would be helpful to learn from the FP, many Medicaid-insured families expressed that FP support could help increase their understanding of their child and promote their own learning about how to best support their child. As one caregiver explained: “Sometimes I don’t understand about the stuff that my son [is] going through, and I got to learn from somebody else, like with the same problem that my son going through” (P3). This sentiment was echoed in content shared by other Medicaid-insured families who described themselves as “not experienced” or “not knowing much” in the area of pediatric anxiety or OCD. Medicaid-insured families also highlighted their desire to learn from the FP in terms of concrete suggestions for how to interact or support their child.

Commercially-insured families also expressed interest in learning from the FP’s lived experience and building connections. However, whereas Medicaid-insured families talked more about long-term relationship building with FPs, commercially-insured families discussed the possibility of FP support differently, with more of a focus on short term support at a specific timepoint, either prior to starting individual therapy or after treatment ended. They emphasized the FP’s potential role in suggesting and connecting to services. One such caregiver shared how they envisioned FP support being helpful: “Maybe if there is a really long wait, maybe they can help bridge the gap. Like before a provider is assigned, maybe a peer-parent can offer some of the services that the family is looking for” (P4). As another commercially-insured family shared about their view of the FP as a service connector: “For me, it’s more a matter of how to help my child, so it’s more a matter of getting to the services for the child than anything else” (P10).

### FP Provider Characteristics

In response to questions about which aspects of an FP’s identity, background, or training might build trust with caregivers, respondents expressed that it would not be important to them to know about the demographic characteristics of the FP, including race, ethnicity, gender identity, or culture. Caregivers instead highlighted the importance of an FP having a child of a similar age and with similar diagnostic characteristics to their own. They expressed that these common characteristics between the FP and the families would make the FP’s lived experience more applicable and any knowledge they had gained around steps taken and services accessed more applicable. As one caregiver shared: “You know, it doesn’t need to be ‘I have an 11-year-old girl who’s gay.’ But, you know, it’s anxiety, social anxiety, and those things in a 6-year-old or a 5-year-old are very different in how you handle them than a 13, 14, 15-year-old” (P9). They also shared how these common child characteristics would help to build rapport. As one caregiver shared, “I just want somebody that’s going through the same thing my child [is] going through ‘cause they will be more understanding and everything” (P3). Another caregiver shared the importance of the commonality of the lived experience specific to child symptoms:

I feel like, potentially, depending on the parent, if you met with someone, you’re going into it knowing you already have something in common and you’re not trying to feel it out. Like are you going to tell me you understand my situation because you kind of felt a little anxious the other day? Or are you going to tell me because you’re walking with your child- you know who can never be left alone like that because they’re so anxious? Like that kind of thing […] lays some early groundwork and help you go a little bit more honestly or deeper with someone faster. (P7)

Medicaid-insured families specifically discussed the importance of trust building with the FP. One caregiver shared the importance of knowing about confidentiality expectations for the FP:

Well it takes a lot for me to trust people. […] It’s just hard for me to trust people like cause I might tell somebody something and then they might tell somebody and stuff, and anything like that. (P3)

Another shared that it would be important to know for how long and in what capacity the FP had been working with our clinic to build trust with them, and that their trust in the clinic would extend to the FP. As they explained it, “I will trust them off of trusting you” (P5).

In addition to the FP’s lived experience, caregivers also expressed that it would be important for FPs to have some knowledge and training related to mental health systems and pediatric anxiety and OCD. The importance of this knowledge and training was often linked back to the FP’s ability to recommend appropriate services. Families often described specific training and knowledge as important but clarified that they did not expect FPs to have the same level of expertise as mental health clinicians. As one caregiver explained: “Yeah, I mean I think knowledge of the issue, basic, but again, not a therapist of, but basic knowledge that they’ve gleaned from dealing with their own issues I think is important” (P9). Another described: “I don’t think they need to be the expert on [anxiety and OCD]. I think they just need to be the expert on getting me to the right expert” (P11). Another caregiver explained a similar sentiment:

I think it, you know, if you’re going to be paired with someone, they should be knowledgeable, so in case you have a question about certain things, they can be able to help you. If they don’t know the answer, then I believe that they should probably know a start to where they can get the answer. (P1)

While caregivers across insurance groups expressed it would be important for the FP to have training and knowledge in system navigation and knowledge about anxiety and OCD, this was expressed more consistently among Medicaid-insured families. This aligned with the previously discussed theme of Medicaid-insured families feeling like they could benefit from increased knowledge and information about how to support a child with anxiety or OCD.

Beyond formal training, many families expressed the importance of the FP’s approach. They expressed the importance of the FP being able to see their perspective and be open-minded. As one caregiver explained:

But I have a thing like when it comes to like statistics and certain training and everything, I believe to keep your training, you know, there, but also keep an open mind, because you haven’t had experience with this particular child. So everyone is different, you know. Everyone takes in things differently, and everyone perceives things differently. So my thing is for them to have their training on one shoulder, but keep an open mind on the other shoulder, because like I said, you’re working with someone that wasn’t in your training or, you know, they didn’t do a study on this particular person. (P1)

Several commercially-insured families expressed the importance of the FP’s having a similar financial background or an understanding of the family’s financial situation. They shared that the importance of this would relate to the FP’s ability to suggest services that were indeed accessible to the family. As one caregiver explained:

I think it’ll be hard to meet with someone who is able to just pay for anything right out of pocket. And especially if they found this amazing thing, and then like it’s like that’s great, I can’t do that. (P7)

### Ways that Clinics can Better Support Families

We also coded themes related to participant suggestions for how clinics can better support caregivers seeking treatment for pediatric anxiety and OCD. We observed no differences here by insurance status. Common suggestions related to the waitlist, which was noted as a primary barrier to care by families. Caregivers recommended hiring more staff to decrease waitlist times and providing additional reminders and updates for families to ensure that they do not forget their appointment during the time between joining the waitlist and an initial intake appointment. Caregivers shared it would have been helpful to get frequent reminders prior to the appointment so it was “fresh in their mind.” As one caregiver shared:

Yeah, long waitlists are never helpful, but I get that staffing shortages are a thing. It’s just sometimes where I’m on several different very long waiting lists [laughs], I’ll get a call and I’ll even forget I was on a waiting list because it’s been a year. So, I think if someone’s on a waiting list to send regular reminders like, “We’re working on it. It’s on the list”, oftentimes if you’re not organized, you just forget which list you’re already on, and so, I think just getting lost sometimes- it feels like it can get lost in the system. And then, suddenly it’s like, “Oh, we have someone available for next week. Oh my god, I’ve been doing service for 6 months somewhere else. What are you talking about? So, like regular communication would be nice. (P11)

Caregivers also recommended additional transportation options, like a ride service, to facilitate access to the clinic. Caregivers also commonly suggested that they receive a list of referrals or connections to other providers who could provide specialized anxiety services. They also suggested ways to increase child engagement prior to initiation of treatment, including clinic-developed handouts they could give to their children to explain treatment, the provision of rewards or incentives, and offering group services for children.

### Quantitative Ratings of Perceptions of Utility of Different Types of FP Support

Quantitative ratings in response to Likert questions about the utility of different types of peer support aligned with qualitative themes about overall positive perceptions of the utility of FP support. Across the six types of possible FP supports queried, quantitative ratings were high with mean ratings above 7 out of 10, with 10 being most helpful. Perceived utility ratings were largely uniform for the FP providing: (1) information and educational support related to child development or anxiety disorders, (2) informational and instructional support on parenting strategies or crisis management, (3) resources for service connection (e.g., respite or transportation), and (4) advocacy support. Ratings of perceived utility were more variable for FP provision of emotional and affirmation support to promote caregiver well-being and support for communication between families and mental health clinicians. Additional quantitative information including illustrative elaborations is provided in Table 2.

Consistent with differences in themes observed by insurance status, Medicaid-insured families rated the potential utility of FP support higher across all categories. Despite the small sample size, these differences were statistically significant for information and educational support related to child development or anxiety disorders (*t*(10) = −2.69, *p* = .023), support for communication between families and mental health clinicians (*t*(9) = −2.99, *p* = .015), providing resources for service connection (*t*(10) = −3.21, *p* = .009), and advocacy support (*t*(10) = −2.26, *p* = .047). See Table 3.

## Discussion

Overall, results indicate that caregivers of anxious youth who did not attend an initial intake appointment at a specialty pediatric anxiety clinic perceived FP support as a potentially useful resource. Caregivers in this sample shared a range of barriers to attending their initial appointment some of which were specific to or amplified for caregivers of children with pediatric anxiety. This includes long waitlist time, difficulty keeping track of where they were on the waitlist, being uncertain about whether to seek treatment, and knowing whether presenting concerns were related to anxiety. Caregivers in the sample highlighted the importance of the FP’s lived experience and how that could help reduce caregiver feelings of isolation. They also shared they were eager to learn from FP’s experiences about how they coped and the decisions they made for their anxious child. They also expressed a clear preference for an FP with shared child diagnostic features and of a similar age as their child. This study is the first to specifically examine the perceived utility of FP support for pediatric anxiety and adds to initial research (Crouch et al., 2019; Frank et al., 2020) suggesting FP support may be well-suited to this population that experiences unique and pervasive barriers to accessing treatment. These findings also extend our understanding of FP support by providing pragmatic recommendations for the development of FP programs tailored to pediatric anxiety and suggest the promise of FP support to address barriers to and promote engagement in care for this population.

Though overall interest in FP support was expressed across the sample, we observed important differences by insurance status. Medicaid-insured families more consistently reported interest in this service for themselves. This was further supported by quantitative results with Medicaid-insured families rating the utility of the different types of FP support higher across categories, with differences between groups reaching statistical significance for most categories queried. Categories rated significantly higher by Medicaid-insured families also mapped onto some of the unique barriers described by Medicaid-insured families. For example, Medicaid-insured families rated the importance of learning from the FP about child development and anxiety disorders, accessing FP support for service connection, and advocacy support within mental health systems significantly higher than commercially-insured families. This maps onto themes uniquely identified among Medicaid-insured families including feeling uncertain about their child’s diagnosis and symptoms, encountering additional structural barriers to attending their first appointment, and emphasis on the importance of FPs having knowledge about how to navigate mental health systems. This also maps onto themes unique to pediatric anxiety related to uncertainty about anxiety diagnosis and symptoms.

Differences in how Medicaid and commercially-insured families discussed their needs and related perceptions of the utility of FP support can be interpreted through the lens of cultural capital (Bourdieu, 1977, 1990). Cultural capital, a term first developed by Pierre Bordieu, broadly refers to how individuals with more financial or educational resources can capitalize on societal resources because of common cultural practices. This, in turn, exacerbates system-level disparities, with individuals who have more resources being more easily able to access resources. We also consider cultural health capital (Dubbin et al., 2013), a derivate of cultural capital. Cultural health specifically refers to how healthcare professionals and patient resources impact communication and provision of care. While we did not specifically measure household income nor did we analyze our themes by educational attainment, we used Medicaid insurance as a proxy variable for access to financial and other resources, given that families must qualify for the program based on income.

Among our Medicaid-insured families, they described more uncertainty about their child’s symptoms and not knowing how to best help their child. They also reported desire for long term relationship building with the FP. This was also reflected in higher quantitative ratings for all categories of FP support as compared to commercially-insured families. Medicaid families also highly rated the importance of FP support for communication between families and mental health clinicians, which may relate back to the concept of cultural health capital impacting communication between providers and families.

In contrast, our commercially-insured families described that given their increased networks and cultural capital, they often had access to resources, including support from others (e.g. through professional or peer networks), leading to the ability to schedule with another provider more quickly. In fact, commercially-insured families often shared about already having access to many of the types of support an FP might deliver through connection to resources and others with similar lived experience. Consistent with this way of conceptualizing caregiver responses, commercially insured families may have other experiences or perspectives that undervalue the utility of FP support. In other words, lower ratings of potential utility may be the result of already having many of their needs met. They also reported less need for long term relationship building with FPs as compared to their Medicaid-insured counterparts and less need for FP support for communication between families and mental health clinicians.

Taken together, FP support may be uniquely suited to the needs of Medicaid insured families seeking anxiety treatment by addressing some of the system-level gaps and additional barriers that Medicaid-insured families face in accessing care (Koob et al., 2023). While many clinics may not be able to directly target structural barriers such as transportation, FP support may be effective in helping caregivers effectively navigate these barriers. Further, previous studies have found that families who have higher levels of overall stress are more likely to utilize FP support (Gopalan et al., 2010; Gyamfi et al., 2010). This suggests that another utility of FP support may be in alleviating caregiver stress, which in turn may increase the caregiver’s ability to address barriers to care. Further, caregivers reported that FP support had the potential to target some of the specific barriers they experienced in accessing anxiety services; notably, gaps in knowledge about anxiety disorders and anxiety-specific treatment as well as the emotional toll associated with this uncertainty. FP support might counteract these barriers either while caregivers are on waitlists for care or as an adjunctive support while in treatment. This is particularly important for this population who we know may be face particularly high barriers in access care to care due to low availability of treatment.

Our study has several practical implications for the development of pediatric anxiety specific FP programs. Differences in themes related to interest in FP support by insurance status indicate that such programs will benefit from flexibility and the ability to tailor the program to specific family needs. Overall, this is consistent with tenets of existing FP models, which allow FPs to work with families to identify their own specific needs (Anthony et al., 2019). While our results suggest that Medicaid-insured families may be interested in building longer-term relationships with an FP, they also suggest the importance of offering shorter-term options to bridge service gaps to be more aligned with the expressed desires of commercially-insured families. Specialized FP programs should allow FPs the flexibility to choose to deliver specific types of support based on family’s expressed needs and preferences. In addition to considering these individual needs and preferences, our results suggest the importance of FPs weaving in their own lived experience and decisions they made for their child while working with families.

Results can guide future efforts to enhance caregiver of anxious youth engagement in initial therapy appointments and inform the development of tailored pediatric anxiety FP programming. FPs might also support with ongoing engagement in mental health treatment once families successfully connect to treatment. Qualitative themes shared by caregivers in our sample suggest that an anxiety-specific FP protocol should include elements such as FPs sharing their own lived experience and how they made decisions related to their child, provision of education about normative versus beyond normative anxiety, distinguishing anxiety from other presenting concerns, and specific education about ranges of types of services available, as well as specific education about exposure therapy for anxiety. Further, FPs should have the flexibility to deliver types of support that were less consistently expressed across caregivers such as support for communication between caregivers and mental health professionals and support for caregiver coping and wellbeing. Our results also suggest the importance of perceived sameness of youth diagnostic characteristics and age between FP and families they serve. While in practice this may be difficult to do, these expressed preferences suggest that potential prioritization of recruiting and training FPs with older children or those with more common anxiety presentations.

This study also has limitations worth noting. While a strength of this study was our intentional focus on reaching caregivers who did not connect with services at our clinic, perceptions of FP utility, particularly once therapy services have been established, may differ in families who were successfully able to connect with services. Further, this study interviewed families with no experience of FP support at our clinic; thus, we were only able to assess perceived utility rather than actual acceptability of FP support. Because caregivers did not have direct experience with working with an FP, they may not have been able to identify all aspects of FP support that would have been helpful. Future studies might specifically query caregivers about a comprehensive list of tasks that FPs can do to support families at various phases of engagement with mental health services, as families may not be aware of all the ways that FPs can support families. This study also occurred within the context and direct aftermath of the COVID-19 pandemic, where demand for mental health services, especially services for children and adolescents, surged (Benton et al., 2022). This resulted in many clinics having long waitlists. As is the case at our clinic, wait times may have shortened since, limiting the generalizability of these findings. Finally, study results are based on a small sample of twelve caregivers. Future studies should aim to see if results, particularly quantitative results, are replicated in a larger sample.

Our study builds on initial work that indicates the potential for FP support to bridge important gaps in our mental health system (e.g., Bearman et al., 2022; Day et al., 2012) and highlights the potential role of specialized FP support for families of anxious youth who might otherwise be unlikely to engage in treatment. Results also suggest that FP support may be helpful in addressing unique barriers specific to pediatric anxiety. Future studies should test the feasibility and acceptability of anxiety-specific FP programs. Future studies should also examine whether FP support for anxious youth is effective in addressing barriers to care and engaging families who encounter significant barriers, such as Medicaid-insured families. More broadly, while FP services are already reimbursable by Medicaid in many states (Schober & Baxter, 2020), our finding that FP support may be particularly acceptable to Medicaid-insured groups highlights the importance of continued efforts to make FP support reimbursable across the country, and at a competitive rate required to ensure sustainment of these programs.

## Supplementary Material

Appendix 1

Appendix 2

**Supplementary information** The online version contains supplementary material available at 10.1007/s10826-025-03173-1.

## Figures and Tables

**Table 1:** Sociodemographic Characteristics of Participants

Sample Characteristics	*n*	%	*M*	*SD*
Age				
Caregiver^a^	9		45	5.72
Child	12		8.33	2.71
Child Gender				
Female	5	41.67		
Male	7	58.33		
Child Insurance Status				
Medicaid	5	41.67		
Employee Insurance Plan	1	8.33		
Other Commercial Insurance	6	50.00		
Caregiver Relationship to Child				
Father	2	16.67		
Mother	9	75.00		
Other	1	8.33		
Caregiver Race				
Asian	1	8.33		
Black or African American	1	8.33		
White	5	41.67		
More than one race	2	16.67		
Not reported	3	25.00		
Caregiver Ethnicity				
Not Hispanic/Latino	8	66.67		
Unknown/Not Reported	4	33. 33		
Caregiver Highest Degree				
Training school (no college)	1	8.33		
Some college	1	8.33		
Associate’s	1	8.33		
Bachelor’s	2	16.67		
Master’s	4	33.33		
Not reported	3	25.00		

a Three caregivers did not report on their age

**Table 2:** Caregiver Ratings of Interest in Receiving Different Types of Family Peer Support

Type of FP Support	M (SD)^a^	Range	Illustrative Elaboration
Information and educational support related to child development or anxiety disorders	8.08 (1.79)	5–10	*“I think understanding it I think a big piece of it is like what’s normal and what pushes beyond that normal. And when to start the process of getting help. I wish I started a little sooner especially if there was going to be a waitlist. So just having that information of like this is- this falls in the range of everybody kind of goes through it, give it a minute, things about separation anxiety, you know like- and then if it gets to this point and we’re still here, then it’s helpful to reach out and get a little help.”(P7)*
Information and instructional support on parenting strategies or crisis management	8.13 (1.71)	6–10	*“No, I mean I think it’s helpful for somebody to tell you like what worked for them or didn’t work for them so parenting strategies are helpful because they can give you ideas, things you have not thought of yet.”(P10)*
Emotional and affirmational support to promote caregiver well being	7.67 (2.35)	3–10	*“I think it’ll help us a lot, like I said, I have been through a lot, so it’s like, the more support, it’s the best for me and my son.”(P3)*
Support for communication between families and mental health clinicians	7.32 (2.82)	2.5–10	*“I give that a 10 because […] having someone there that knows what you’re going through and kind of has intel […] on what you want to ask, or your concerns, is great for times like this[…].”(P1)*
Providing resources for service connection (e.g., respite or transportation)	8.17 (1.53)	5–10	*“Um, 9. I think those are really big barriers for entry for some families.”(P4)*
Advocacy support (e.g., information about how to navigate mental health systems, parent rights, how to advocate for their child	8.58 (1.24)	8–10	*“Yeah, I just think the system is so complicated in general and everyone should absolutely know like how [..]they kind of just need to advocate for their own kids. Otherwise, they’re not going to get the care they need. So, I think everyone needs [it].”(P11)*

a Scale ratings from 1=not helpful to 10=helpful

**Table 3:** Ratings of Interest in Receiving Different Types of Family Peer Support Stratified by Insurance Type

	Medicaid		Commercial Insurance		*n*	*df*	*t*	*P*
		
	M (SD)^a^	Range	M (SD)^a^	Range				
Information and educational support related to child development or anxiety disorders	9.4 (0.89)	8–10	7.14 (1.70)	5–9	12	10	−2.689	.023^b^
Information and instructional support on parenting strategies or crisis management	8.3 (1.57)	6–10	8 (1.91)	6–10	12	10	−0.287	.780
Emotional and affirmational support to promote caregiver well being	9 (1.41)	7–10	6.71 (2.50)	3–10	12	10	−1.831	.097
Support for communication between families and mental health clinicians	9.4 (1.34)	7–10	5.58 (2.56)	2.5–9	11	9	−2.993	.015^b^
Providing resources for service connection (e.g., respite or transportation)	9.4 (0.89)	8–10	7.29 (1.25)	5–9	12	10	−3.213	.009^b^
Advocacy support (e.g., information about how to navigate mental healthsystems, parent rights, how to advocate for their child)	9.4 (0.89)	8–10	8 (1.15)	7–10	12	10	−2.259	.047^b^

a Scale ratings from 1 = not helpful to 10 = helpful

b *p* < 0.005

## Highlights

- Family Peer (FP) support shows promise in the broader child mental health literature but has not yet been developed or tested specifically for caregivers of anxious youth.
- This study interviewed caregivers of anxious youth who scheduled but did not attend an initial appointment at a specialty pediatric anxiety clinic.
- Results discuss barriers and facilitators to attending the appointment, as well as perceptions of the potential utility of FP support.
- FP support was deemed acceptable and of interest to caregivers in the sample, with differences observed by insurance type.
- We discuss how these findings inform the development of a pediatric anxiety specific FP program at our clinic as well as the broader policy implications of these findings.

## References

[R1] Anthony BJ, Serkin C, Kahn N, Troxel M, & Shank J (2019). Tracking progress in peer-delivered family-to-family support. Psychological Services, 16(3), 388–401. 10.1037/ser0000256. 30382742

[R2] Bearman SK, Jamison JM, Lopez MA, Baker NM, & Sanchez JE (2022). Testing the impact of a peer-delivered family support program: A randomized clinical effectiveness trial. Psychiatric Services (Washington, D.C.), 73(7), 752–759. 10.1176/appi.ps.202100278. 35042370

[R3] Benton T, Njoroge WFM, & Ng WYK (2022). Sounding the alarm for children’s mental health during the COVID-19 pandemic. JAMA Pediatrics, 176(4), e216295 10.1001/jamapediatrics.2021.6295. 35129603

[R4] Bitsko RH (2022). Mental health surveillance among children—United States, 2013–2019. MMWR Supplements, 71, 1–42. 10.15585/mmwr.su7102a1. PMC8890771 35202359

[R5] Bourdieu P. (1977). Outline of a theory of practice (Nice R, Trans.). (Cambridge University Press. 10.1017/CBO9780511812507

[R6] Bourdieu P. (1990). The logic of practice. (Stanford University Press.

[R7] Carrillo JE, Carrillo VA, Perez HR, Salas-Lopez D, Natale-Pereira A, & Byron AT, (2011). Defining and targeting health care access barriers. Journal of Health Care for the Poor and Underserved, 22(2), 562–575. 10.1353/hpu.2011.0037. 21551934

[R8] Chavira DA, Bantados B, Rapp A, Firpo-Perretti YM, Escovar E, Dixon L, Drahota A, & Palinkas LA (2017). Parent-reported stigma and child anxiety: A mixed methods research study. Children and Youth Services Review, 76, 237–242. 10.1016/j.childyouth.2017.03.013. 29576669 PMC5860669

[R9] Clarke V, & Braun V (2017). Thematic analysis. The Journal of Positive Psychology, 12(3), 297–298. 10.1080/17439760.2016.1262613.

[R10] Conn A-M, Szilagyi MA, Alpert-Gillis L, Webster-Stratton C, Manly JT, Goldstein N, & Jee SH (2018). Pilot randomized controlled trial of foster parent training: A mixed-methods evaluation of parent and child outcomes. Children and Youth Services Review, 89, 188–197. 10.1016/j.childyouth.2018.04.035.

[R11] Crouch L, Reardon T, Farrington A, Glover F, & Creswell C (2019). “Just keep pushing”: Parents’ experiences of accessing child and adolescent mental health services for child anxiety problems. Child: Care, Health and Development, 45(4), 491–499. 10.1111/cch.12672. 30990911

[R12] Day C, Michelson D, Thomson S, Penney C, & Draper L (2012). Evaluation of a peer led parenting intervention for disruptive behaviour problems in children: Community based randomised controlled trial. BMJ, 344, e1107 10.1136/bmj.e1107. 22416059

[R13] Deacon BJ, Farrell NR, Kemp JJ, Dixon LJ, Sy JT, Zhang AR, & McGrath PB (2013). Assessing therapist reservations about exposure therapy for anxiety disorders: The therapist beliefs about exposure scale. Journal of Anxiety Disorders, 27(8), 772–780. 10.1016/j.janxdis.2013.04.006. 23816349

[R14] Dubbin LA, Chang JS, & Shim JK (2013). Cultural health capital and the interactional dynamics of patient-centered care. Social Science & Medicine, 93, 10.1016/j.socscimed.2013.06.014 PMC3887515 23906128

[R15] Feinberg E, Abufhele M, Sandler J, Augustyn M, Cabral H, Chen N, Diaz Linhart Y, Cesar Levesque Z, Aebi M, & Silverstein M (2016). Reducing disparities in timely autism diagnosis through family navigation: results from a randomized pilot trial. Psychiatric Services, 67(8), 912–915. 10.1176/appi.ps.201500162. 27133722

[R16] Feinberg E, Augustyn M, Broder-Fingert S, Bennett A, Weitzman C, Kuhn J, Hickey E, Chu A, Levinson J, Sandler Eilenberg J, Silverstein M, Cabral HJ, Patts G, Diaz-Linhart Y, Fernandez-Pastrana I, Rosenberg J, Miller JS, Guevara JP, Fenick AM, & Blum NJ (2021). Effect of family navigation on diagnostic ascertainment among children at risk for autism: a randomized clinical trial from DBPNet. JAMA Pediatrics, 175(3), 243–250. 10.1001/jamapediatrics.2020.5218. 33427861 PMC7802008

[R17] Frank HE, Becker-Haimes EM, & Kendall PC (2020). Therapist training in evidence-based interventions for mental health: A systematic review of training approaches and outcomes. Clinical Psychology: Science and Practice, 27(3), e12330 10.1111/cpsp.12330. PMC8174802 34092941

[R18] Gopalan G, Goldstein L, Klingenstein K, Sicher C, Blake C, & McKay MM (2010). Engaging families into child mental health treatment: updates and special considerations. Journal of the Canadian Academy of Child and Adolescent Psychiatry, 19(3), 182–196. 20842273 PMC2938751

[R19] Gyamfi P, Walrath C, Burns BJ, Stephens RL, Geng Y, & Stambaugh L (2010). Family education and support services in systems of care. Journal of Emotional and Behavioral Disorders, 18(1), 14–26. 10.1177/1063426609333891.

[R20] Hansen AS, Telléus GK, Mohr-Jensen C, & Lauritsen MB (2021). Parent-perceived barriers to accessing services for their child’s mental health problems. Child and Adolescent Psychiatry and Mental Health, 15(1), 4 10.1186/s13034-021-00357-7. 33514400 PMC7847149

[R21] Hennink MM, Kaiser BN, & Marconi VC (2017). Code saturation versus meaning saturation: how many interviews are enough? Qualitative Health Research, 27(4), 591–608. 10.1177/1049732316665344. 27670770 PMC9359070

[R22] Higa-McMillan C, Kotte A, Jackson D, & Daleiden EL (2017). Overlapping and non-overlapping practices in usual and evidence-based care for youth anxiety. The Journal of Behavioral Health Services & Research, 44(4), 684–694. 10.1007/s11414-016-9502-2. 26945583

[R23] Hoagwood KE (2005). Family-based services in children’s mental health: A research review and synthesis. Journal of Child Psychology and Psychiatry, 46(7), 690–713. 10.1111/j.1469-7610.2005.01451.x. 15972066

[R24] Hofmann SG, Asnaani A, Vonk IJJ, Sawyer AT, & Fang A (2012). The efficacy of cognitive behavioral therapy: A review of meta-analyses. Cognitive Therapy and Research, 36(5), 427–440. 10.1007/s10608-012-9476-1. 23459093 PMC3584580

[R25] Ireys HT, & Sakwa DD (2006). Building family-to-family support programs: Rationale, goals, and challenges. Focal Point, 20, 10–14.

[R26] Jamison JM, Fourie E, Siper PM, Trelles MP, George-Jones J, Buxbaum Grice A, Krata J, Holl E, Shaoul J, Hernandez B, Mitchell L, McKay MM, Buxbaum JD, & Kolevzon A (2017). Examining the efficacy of a family peer advocate model for black and hispanic caregivers of children with autism spectrum disorder. Journal of Autism and Developmental Disorders, 47(5), 1314–1322. 10.1007/s10803-017-3045-0. 28168677

[R27] Koob C, Stuenkel M, Gagnon RJ, Griffin SF, & Sease K (2023). Identifying risk factors associated with repeated referrals within a pediatric navigation program. Journal of Community Health, 48(6), 1044–1051. 10.1007/s10900-023-01274-w. 37658945

[R28] Kutash K, Duchnowski AJ, Green AL, & Ferron JM (2011). Supporting parents who have youth with emotional disturbances through a parent-to-parent support program: A proof of concept study using random assignment. Administration and Policy in Mental Health, 38(5), 412–427. 10.1007/s10488-010-0329-5. 21136148

[R29] Meier SM, & Deckert J (2019). Genetics of anxiety disorders. Current Psychiatry Reports, 21(3), 16 10.1007/s11920-019-1002-7. 30826936

[R30] Merikangas KR, He J, Burstein M, Swendsen J, Avenevoli S, Case B, Georgiades K, Heaton L, Swanson S, & Olfson M (2011). Service utilization for lifetime mental disorders in U.S. adolescents: Results of the National Comorbidity Survey–adolescent supplement (NCS-A). Journal of the American Academy of Child & Adolescent Psychiatry, 50(1), 32–45. 10.1016/j.jaac.2010.10.006. 21156268 PMC4408275

[R31] Muroff J, & Ross A (2011). Social disability and impairment in childhood anxiety. In McKay D & Storch EA (Eds.), Handbook of Child and Adolescent Anxiety Disorders (pp. 457–478). Springer. 10.1007/978-1-4419-7784-7_31

[R32] Owens PL, Hoagwood K, Horwitz SM, Leaf PJ, Poduska JM, Kellam SG, & Ialongo NS (2002). Barriers to children’s mental health services. Journal of the American Academy of Child & Adolescent Psychiatry, 41(6), 731–738. 10.1097/00004583-200206000-00013. 12049448

[R33] Reardon T, Harvey K, Baranowska M, O’Brien D, Smith L, & Creswell C (2017). What do parents perceive are the barriers and facilitators to accessing psychological treatment for mental health problems in children and adolescents? A systematic review of qualitative and quantitative studies. European Child & Adolescent Psychiatry, 26(6), 623–647. 10.1007/s00787-016-0930-6. 28054223 PMC5446558

[R34] Reardon T, Harvey K, Young B, O’Brien D, & Creswell C (2018). Barriers and facilitators to parents seeking and accessing professional support for anxiety disorders in children: Qualitative interview study. European Child & Adolescent Psychiatry, 27(8), 1023–1031. 10.1007/s00787-018-1107-2. 29372331 PMC6060962

[R35] Saavedra LM, Silverman WK, Morgan-Lopez AA, & Kurtines WM (2010). Cognitive behavioral treatment for childhood anxiety disorders: Long-term effects on anxiety and secondary disorders in young adulthood. Journal of Child Psychology and Psychiatry, 51(8), 924–934. 10.1111/j.1469-7610.2010.02242.x. 20345838

[R36] Salloum A, Johnco C, Lewin AB, McBride NM, & Storch EA (2016). Barriers to access and participation in community mental health treatment for anxious children. Journal of Affective Disorders, 196, 54–61. 10.1016/j.jad.2016.02.026. 26901657

[R37] Schober M, & Baxter K (2020). Medicaid funding for family and youth peer support programs in the United States. University of Maryland School of Social Work. https://theinstitute.umaryland.edu/media/ssw/institute/national-center-documents/PSM2020.pdf

[R38] Singer GHS, Marquis J, Powers LK, Blanchard L, Divenere N, Santelli B, Ainbinder JG, & Sharp M (1999). A multi-site evaluation of parent to parent programs for parents of children with disabilities. Journal of Early Intervention, 22(3), 217–229.

[R39] Whiteside SPH, Sim LA, Morrow AS, Farah WH, Hilliker DR, Murad MH, & Wang Z (2020). A Meta-analysis to guide the enhancement of cbt for childhood anxiety: exposure over anxiety management. Clinical Child and Family Psychology Review, 23(1), 102–121. 10.1007/s10567-019-00303-2. 31628568 PMC9005063

[R40] Wolitzky-Taylor K, Chung B, Bearman SK, Arch J, Grossman J, Fenwick K, Lengnick-Hall R, & Miranda J (2019). Stakeholder perceptions of the barriers to receiving and delivering exposure-based cognitive behavioral therapy for anxiety disorders in adult community mental health settings. Community Mental Health Journal, 55(1), 83–99. 10.1007/s10597-018-0250-z. 29508179 PMC6123294

[R41] Zifkin C, Montreuil M, Beauséjour M-È, Picard S, Gendron-Cloutier L, & Carnevale FA (2021). An exploration of youth and parents’ experiences of child mental health service access. Archives of Psychiatric Nursing, 35(5), 549–555. 10.1016/j.apnu.2021.06.006. 34561072

