# Supplementary material for: Caregiver Perceptions of the Potential Utility of a Specialized Family Peer Program for Anxious Youth
Source: J Child Fam Stud. Author manuscript; Available in PMC 2026 Jun 19. (PMC13278483; doi:10.1007/s10826-025-03173-1)
Supplement: Appendix 1 [file NIHMS2180642-supplement-Appendix_1.docx]

**Appendix 2**

**INTAAKE Qualitative Coding Guide**

| **Code** | **Definition** |
| --- | --- |
| **Barriers to Accessing Mental Health Services** | Code when participant mentions factors that impeded their access to or engagement with mental health services. |
| **Facilitators to Accessing Mental Health Services** | Code when participant mentions factors that facilitated their accessing mental health services or if something was explicitly noted as not a barrier to their ability to receive care. This involves factors that are already in place. Do not code if parent simply responds “No” to a question asking if something is a barrier.  ***Item Distinction:*** *This is distinct from* ***Suggestions for Improvement*** *in that this item describes things already in place that facilitated access.*  *e.g.* ***Suggestion for Improvement:*** *“It’d be easier to make appointments if I had a case worker who could arrange transportation”*  *e.g.* ***Facilitators to Accessing Mental Health Services****: “Having access to the case worker made getting to my appointment easier because they could help with transportation.”*  ***Item Distinction:*** *This is distinct from* ***Reasons for Seeking Mental Health Treatment*** *which identifies the reason that someone sought out mental health treatment.* |
| **Perceptions of Telehealth** | Code when participant discusses perceptions of telehealth, regardless of whether they directly relate to their child’s ability to access care.  ***Item Notes****: May be double coded* ***with Facilitators to Accessing Mental Health Services*** *or* ***Barriers to Accessing Mental Health Services****.* |
| **Past Experiences Working with Mental Health Navigator** | Code responses about past experiences (or lack thereof) of working with a mental health navigator (e.g., case worker, peer specialist, anyone who helped with navigating mental health services)  ***Item Notes:*** *This item does not include past experiences with mental health services in general, just navigators as outlined above.* |
| **Initial Reactions about Peer Parent** | Code responses about initial reaction towards meeting with a peer parent.  ***Item Notes:*** *This item will often be double coded with* ***Helpful Aspects of Meeting with a Peer Parent*** *or* ***Unhelpful Aspects of Meeting with a Peer Parent.*** |
| **Helpful Aspects of Meeting with a Peer Parent** | Code when participant identifies aspects of meeting with a peer parent that would be helpful for either themselves or other parents.  ***Item Notes:*** *Will likely include responses to question #6 (Initial reactions) and question #8 (Types of support interested in receiving).*  ***Item Distinction:*** *This differs from* ***Important Aspects of Peer Parent’s Identity*** *in that we are code about the content of FPA support or the experience of receiving this support as opposed to characteristics of the person providing the support.* |
| **Unhelpful Aspects of Meeting with a Peer Parent** | Code when participant identifies aspects of meeting with a peer parent that would be unhelpful for either themselves or other parents. Will likely include responses to question #6 (Initial reactions). |
| **FP Provider Characteristics -- Important Aspects of Peer Parent’s Identity** | Code aspects of a peer parent’s identity/ who they are that the participant identifies as important for gaining trust.  ***Item Notes:*** *Do not double code* ***Helpful Aspects*** *if participant is further expanding on why this aspect is important (e.g. “And them having some experience, knowledge about that could help me avoid some pitfalls if any, you know.”*  ***Item Distinction:*** *This differs from* ***Important Aspects of Peer Parent’s Support*** *in that we are coding characteristics of the person providing FPA support versus as opposed to content of FPA support or the experience of receiving this support.* |
| **FP Provider Characteristics -- Unimportant/Not Relevant Aspects of Peer Parent’s Identity** | Code aspects of a peer parent’s identity that the participant specifically identifies as not being important or relevant for gaining trust. Include this code if someone answers “No” to a question about whether a specific aspect is important.  ***Item Notes:*** *Do not double code* ***Unhelpful Aspects*** *if participant is further expanding on why this aspect is not important to them.* |
| **Ways that Clinics Can Better Support Families**  **-- Suggestions for Improvements** | Code responses that provide suggestions of how mental health clinics in general or the mental health field can improve their support of families. These are things the participant wishes or want that they don’t already have access to.  ***Item Distinction:*** *This is distinct from* ***Facilitators to Accessing Mental Health Services*** *in that this item describes things NOT already in place that would facilitate access from the respondent’s perspective*  *e.g.* ***Suggestion for Improvement:*** *“It’d be easier to make appointments if I had a case worker who could arrange transportation”*  *e.g.* ***Facilitators to Accessing Mental Health Services****: “Having access to the case worker made getting to my appointment easier because they could help with transportation.”* |
| **Ways that Clinics Can Better Support Families**  **-- Types of Support Other than Peer Parent Identified** | Code when participant identifies types of support that would be helpful that is NOT a peer parent or individual mental health treatment. |
| **Perceptions of helpfulness of Information or Educational Support (Question 9)** | Code response to question 9 if participant adds any information to their response aside from the numerical response. |
| **Perceptions of helpfulness of Information on Parenting Strategies or Crisis Management (Question 10)** | Code response to question 10 if participant adds any information to their response aside from the numerical response. |
| **Perceptions of helpfulness of Emotional of Affirmational Support (Question 11)** | Code response to question 11 if participant adds any information to their response aside from the numerical response. |
| **Perceptions of helpfulness of Promoting Communication Between Families and Mental Health Clinicians (Question 12)** | Code response to question 12 if participant adds any information to their response aside from the numerical response. |
| **Perceptions of helpfulness of Connection to Services (Question 13)** | Code response to question 13 if participant adds any information to their response aside from the numerical response. |
| **Perceptions of helpfulness of Advocacy Support (Question 14)** | Code response to question 14 if participant adds any information to their response aside from the numerical response. |
