# Supplementary material for: Caregiver Perceptions of the Potential Utility of a Specialized Family Peer Program for Anxious Youth
Source: J Child Fam Stud. Author manuscript; Available in PMC 2026 Jun 19. (PMC13278483; doi:10.1007/s10826-025-03173-1)
Supplement: Appendix 2 [file NIHMS2180642-supplement-Appendix_2.docx]

Appendix 1

**Semi-Structure Qualitative Interview**

*Note that this interview is intended to be a guide only – interviewer should ask some or all of these questions depending on what the interviewee shares*

**Introduction**

*Thank you for agreeing to participate in the study. Our goal is to learn from parents like you about how we can improve access to services for families seeking mental health treatment for their child’s anxiety or related disorders like obsessive-compulsive disorder. We are particularly interested in learning your perspectives about things that can get in the way of or help with receiving services and what clinics like ours here at [our clinic] can do better to support families. We reached out to you and your family specifically as our records indicate you reached out to inquire about an appointment with us but did not ultimately complete an intake evaluation. We understand there are many reasons that a family might not end up proceeding with the first formal appointment, and we are interested in understanding these reasons from your perspective. Your responses will help us develop strategies to improve access to mental health services for families like yours. Your answers will not affect your ability to receive future mental health services at [our clinic] or any other program. We will record this interview and keep everything you share fully confidentially, as allowable by law. If we hear information that might compromise a child’s safety, we may not be able to keep that information confidential. If this came up, we would discuss the next steps with you. There are no right or wrong answers, and you can choose not to answer any questions or can stop the interview at any time.*

*Do you have any questions before we get started?*

1. *As a reminder, you originally reached out to us for an appointment at [our clinic] in* ***MONTH/YEAR.*** *Based on our records, you had initially shared during our* ***[SELECT PHONE CALL OR INITIAL ONLINE SCREENER]*** *that you were seeking services for your child’s* ***SYMPTOMS/PRESENTING PROBLEM.*** *Please note that we do not have any other clinical information that may have been shared with [our clinic] other than this basic information. We first were able to offer you an intake appointment in* ***MONTH/YEAR,*** *but you were unable to attend. Would you be willing to share what influenced your ability or decision to not complete the appointment in* ***MONTH****?*
2. Are there any other factors or circumstances that influenced your ability or decision not to attend/complete the appointment?

*Potential Probes:*

*We know there are lots of things that can influence one's decision too, such as (name ones not addressed). Did any of these factors influence your ability or decision to attend?*

- 1. *past experiences with mental health services*
  2. *symptoms your child was experiencing*
  3. *your own thoughts or feelings*
  4. *your friends’ perception of mental health services*
  5. *your family’s perception of mental health services*
  6. *your access to resources, like travel*
  7. *balancing multiple services/ appointments*

1. *If not already answered above:* Were there any components related to our organization, PATCH, in particular that impacted your ability or decision not to attend the appointment?

*Potential Probes:*

- 1. *Location*
  2. *Communication*
  3. *Availability of telehealth*

1. What do you wish mental health clinics, including [our clinic], would do to make it easier for you to attend?

*Thank you for sharing all this information. One strategy we are considering to better support families in accessing treatment services is to develop a family peer service that could help support parents, or a “peer parent”. This peer parent is someone who has lived experiences with the populations they are helping. In this case, the peer parent would be the parent of a child with anxiety or OCD who understands how to access and benefit from anxiety mental health services and has special training in how to support other parents or caregivers. We are interested in your thoughts about the potential utility of connecting parents interested in anxiety or OCD-focused mental health services for their child with a peer parent. We would also like to know your thoughts on what kind of support might be most helpful for a peer parent to provide.*

1. What, if any, experiences have you had working with a case worker, peer specialist, or someone who helped you navigate mental health services with any of your children?
2. Could you tell me your initial reaction towards meeting with a peer parent?

*Potential Probes:*

- 1. *What would be most helpful about meeting with a peer parent?*
  2. *If you feel you can speak to this, how helpful or unhelpful do you think it would be for other parents you know to meet with a peer parent?*

*6c. [SKIP IF THEY SAY PEER PARENT COULD BE HELPFUL]If participant indicates they do NOT think it would be helpful or are not interested, ask:*

*What types of support, if any, might you be interested in to help you navigate or connect with services?*

1. Aspects of a peer parents’ identity, background, or training might be important to different individuals in making them feel like they can trust them. If you (**if family expressed disinterest in peer parents, add “**or another parent”) were to meet with a peer parent, what would be some aspects of who they are that you would want to know about?

*Potential Probes:*

- 1. *the cultural background of the individual*
  2. *the racial/ethnic identity of the individual*
  3. *the gender identity of the individual*
  4. *the symptoms their child experienced or is experiencing*
  5. *other child characteristics (e.g., age, gender identity)*
  6. *types of training they have received*
  7. *knowledge about anxiety and OCD*
  8. *knowledge about mental health systems*
  9. *Some families may want a peer parent to be from a similar income bracket or of similar economic or financial background to best be able to relate to them. How important would that be to you?*

1. If you were meeting with a peer parent, what types of support or help would you be interested in receiving?

*If respondent expresses uncertainty, can say “I actually have a list of some common things…” and move to next section*

*I want to share some common things peer parents can do to support other parents, caregivers, or families. I would like to get your opinion on how helpful or unhelpful each component might be for you when seeking mental health services for youth with anxiety or related disorders like obsessive compulsive disorder. For the last section of this interview, I would like you to rate the following on a scale of 1-10 where 1 is unhelpful and 10 is most helpful.*

1. Providing informational and educational support, such as teaching families about child development or how anxiety disorders present in kids

Response: ________________

Is there anything you would like to share about why you gave this response?

1. Providing information and instruction on parenting strategies or crisis management strategies

Response: ________________

Is there anything you would like to share about why you gave this response?

1. Providing emotional and affirmational support, and promoting caregiver well being

Response: ________________

Is there anything you would like to share about why you gave this response?

1. Helping to promote communication between families and mental health clinicians

Response: ________________

Is there anything you would like to share about why you gave this response?

1. Providing resources for connection to services including resources for respite or short term care and transportation

*If participant asks for clarification about respite:*

*Respite care is short-term relief for primary caregivers that can be provided at home or within a healthcare facility, which can give the primary caregiver a break from the stress of caregiving.*

Response: ________________

Is there anything you would like to share about why you gave this response?

1. Advocacy support, such as informing caregivers about how to navigate mental health systems, discussing parents’ rights and how to advocate for themselves and their child

Response: ________________

Is there anything you would like to share about why you gave this response?

*We are almost done with our interview. Before we finish, I just want to ask a few more questions.*

1. Is there any other advice you would share with us about how a peer parent could be most helpful for families seeking treatment for children with anxiety or obsessive-compulsive disorder?
2. Is there anything else you would like to share with us about how clinics like [our clinic] can do a better job supporting families?
